# Two for the Price of One: A Neuroprotective Chaperone Kit within NAD Synthase Protein NMNAT2

**DOI:** 10.1371/journal.pbio.1002522

**Published:** 2016-07-25

**Authors:** Angela Lavado-Roldán, Rafael Fernández-Chacón

**Affiliations:** Instituto de Biomedicina de Sevilla (IBiS) HUVR/CSIC/Universidad de Sevilla, Departamento de Fisiología Médica y Biofísica and CIBERNED, Seville, Spain

## Abstract

One of the most fascinating properties of the brain is the ability to function smoothly across decades of a lifespan. Neurons are nondividing mature cells specialized in fast electrical and chemical communication at synapses. Often, neurons and synapses operate at high levels of activity through sophisticated arborizations of long axons and dendrites that nevertheless stay healthy throughout years. On the other hand, aging and activity-dependent stress strike onto the protein machineries turning proteins unfolded and prone to form pathological aggregates associated with neurodegeneration. How do neurons protect from those insults and remain healthy for their whole life? Ali and colleagues now present a molecular mechanism by which the enzyme nicotinamide mononucleotide adenylyltransferase 2 (NMNAT2) acts not only as a NAD synthase involved in axonal maintenance but as a molecular chaperone helping neurons to overcome protein unfolding and protein aggregation.

## Abstract 2

This Primer examines recent research showing that a brain enzyme known to ameliorate excitoxicity and axonal degeneration also acts as a molecular chaperone to prevent protein aggregation.

Throughout life, neurons need to overcome multiple types of internal and external attacks that can lead to neuronal damage and ultimately to neuronal death. Those insults happen as a consequence of physiological situations such as high neural activity and aging or are related to neurodegenerative diseases and nerve injury. Within neurons, axons and synapses are particularly vulnerable. Certain neurons, such as some motorneurons, receive half a million of nerve impulses daily [1], imposing a significant stress burden on synaptic proteins and axons. At synapses, for example, the cascade of interacting SNARE proteins mediating neurotransmitter release requires molecular chaperones to support the continuous cycle of folding and unfolding of reactive proteins [2,3]. Molecular chaperones assist protein folding, promote refolding of stress-denatured proteins, and prevent and disrupt the improper interactions that lead to protein aggregation [4]. Interestingly, the removal of a synaptic cochaperone such as Cysteine String Protein-α (CSP-α) leads to activity-dependent synaptic degeneration in mice [5–7], and mutations in the gene *DNAJC5*, coding for CSP-α in humans, cause adult onset autosomal-dominant neuronal ceroid lipofuscinosis, a devastating neurodegenerative disease in young adults [8].

Axons, on the other hand, are particularly susceptible to traumatic damage because of their extended lengths. Upon mechanical brain injury, diffuse axonal damage in brain and spinal cord impacts neuronal function and promotes neurodegeneration [9]. A well-studied model of axonal degeneration is the so-called Wallerian degeneration, the process that leads to progressive fast axonal disappearance upon a distal axonal severing [10]. The study of a mouse mutant that displays an intriguingly delayed Wallerian degeneration has provided important advances to understand mechanisms of axonal protection [10]. Wallerian degeneration slow (WldS) mice bear a gain-of-function mutation that increases activity of the nicotinamide mononucleotide adenylyltransferase 1 (NMNAT1) enzyme. In mammals, there are three isoforms on NMNAT (1–3), and all of them enable the synthesis of nicotinamide adenine dinucleotide (NAD+), an important electron carrier in energy metabolism and cell signaling. Upon axonal damage, the loss of NAD+ would precipitate energetic failures responsible for structural axonal degeneration. Increased NMNAT1 activity protects axons by ameliorating the fast depletion of NAD+ in WldS mice [9,10]. Axonal degeneration is also triggered by the loss of the cytosolic protein NMNAT2, the most labile of all NMNAT isoforms, which vanishes soon after axon injury [11]. Interestingly, the neuroprotective action of NMNATs goes beyond their NAD synthase enzymatic activity because they act as molecular chaperones [12]. Initial studies demonstrating the chaperone function of NMNATs showed that loss of *Drosophila* NMNAT (dNMNAT) caused rapid and severe neurodegeneration that, interestingly, could be rescued by enzymatically inactive dNMNAT [13]. Furthermore, dNMNAT overexpression in flies turned out to be protective against spinocerebellar ataxia 1 (SCA1)-induced neurodegeneration, a disorder characterized by the formation of aggregates of misfolded proteins [14]. Those seminal studies unveiled the molecular chaperone function of dNMNAT independently of the NAD synthase function [13,14].

In vertebrates, the isoform NMNAT2, highly expressed in the brain, functions as a cytosolic protein bound to Golgi-derived vesicles through the palmitoylation of several cysteine residues [15]. NMNAT2 seems to be essential for axonal growth or maintenance during embryogenesis [16]. Indeed, homozygous mutant mice lacking NMNAT2 show a severe phenotype at the peripheral nervous system characterized by a reduction in spinal motorneurons, sensory neurons of the dorsal root ganglia, and absence of axons in the hind limb [16]. Interestingly, specific depletion of NMNAT2 induces Wallerian degeneration of intact axons that cannot be prevented by other NMNAT isoforms [11]. Those studies settled a crucial role of NMNAT2 in axon development and maintenance. Furthermore, similar to what was previously described for dNMNAT, a recent study has pinpointed a molecular chaperone role for NMNAT2 to regulate protein homeostasis in neurodegeneration-related scenarios. This study showed that overexpression of NMNAT2 in the brain reduced pathological signs of neurodegeneration in rTg4510 mice, a transgenic model of frontotemporal dementia with parkinsonism linked to chromosome 17 (FTDP-17), which expresses the human protein Tau with the missense mutation P301L [17]. These results indirectly supported the notion that NMNAT2 could be acting as a molecular chaperone to protect against neurodegeneration by, however, unknown mechanisms [17].

Now, Ali et al. [18] have carried out a detailed study that presents associations of NMNAT2 with cognitive decline and Alzheimer disease (AD). By analyzing the transcriptome of cortical samples from a cohort of 541 deceased subjects, the authors found a positive relationship between the levels of *NMNAT2* mRNA and global cognition scores, which does not occur for NMNAT1. In addition, in a different cohort, the authors found a subtle decrease of NMNAT2 mRNA, in contrast to normal levels of NMNAT1, in brains from AD patients. Strikingly, protein analysis in AD brains revealed an abnormal shift of NMNAT2 from the soluble to the insoluble biochemical fractions. Those results suggest that high NMNAT2 levels might prevent cognitive decline.

Next, Ali et al. set up a cell-based assay to explore the potential chaperone activity of NMNAT2 upon heat shock-induced protein denaturation. In that assay, NMNAT2 was not only capable of preventing heat-induced protein denaturation but could also promote refolding of denatured proteins. Both effects were independent of NAD synthase activity. However, while the holdase activity that prevents denaturation was independent of ATPase activity, the foldase activity that refolded proteins was critically dependent on the NMNAT2 C-terminal ATP binding site. The authors reinforced those notions using the rTg4510 mice. They demonstrated that NMNAT2’s ability to clear the proteotoxic hyperphosphorylated human Tau form (p-hTau) in vivo required the intact ATP binding site, and it was independent of NAD synthase, which suggested that the foldase activity was required.

To investigate the molecular mechanism underlying the NMNAT2 chaperone function, Ali et. al used a cell line that overexpresses a pathogenic version of human Tau. Immunoprecipitation of NMNAT2 from these cells uncovered that NMNAT2 complexes with heat shock protein 90 (HSP90), an abundant and ubiquitous molecular chaperone that plays an essential role in maintaining protein homeostasis. Interestingly, HSP90 was required for NMNAT2 to act as a foldase to reduce Tau aggregation, while NMNAT2’s holdase activity was independent of HSP90. Thoroughly, the authors corroborated the chaperone function of the NMNAT2-Hsp90 tandem in a different cellular assay based on the pathological aggregation of the protein ataxin-1, the protein responsible for neurodegeneration in SCA1.

Finally, the authors took advantage of knock-out (KO) mice lacking NMNAT2, which, though they die at birth, are useful for neuronal cultures. Curiously, the viability of the cultures decreased as they matured and formed synapses, and it worsened when protein degradation was experimentally inhibited to promote proteotoxicity or when neuronal excitation was increased to promote excitotoxicity. Interestingly, Ali et. al showed that the ATP-dependent foldase activity was required to protect from proteotoxicity, while the NAD synthase enzymatic activity was required to prevent excitotoxicity (Fig 1). Those observations remarked the dual role of NMNAT2 to maintain neuronal health in a context-dependent manner. Interestingly, NMNAT2 is enriched in synaptic terminals [15,19], and Ali et al. found significant reductions in the levels of presynaptic protein in neuronal cultures and brain extracts from NMNAT2-KO and heterozygous mice. Those observations suggest that NMNAT2 might be important for synaptic maintenance, although those changes in synaptic proteins could also be, in part, secondary to the axonal deterioration.

**Fig 1:**
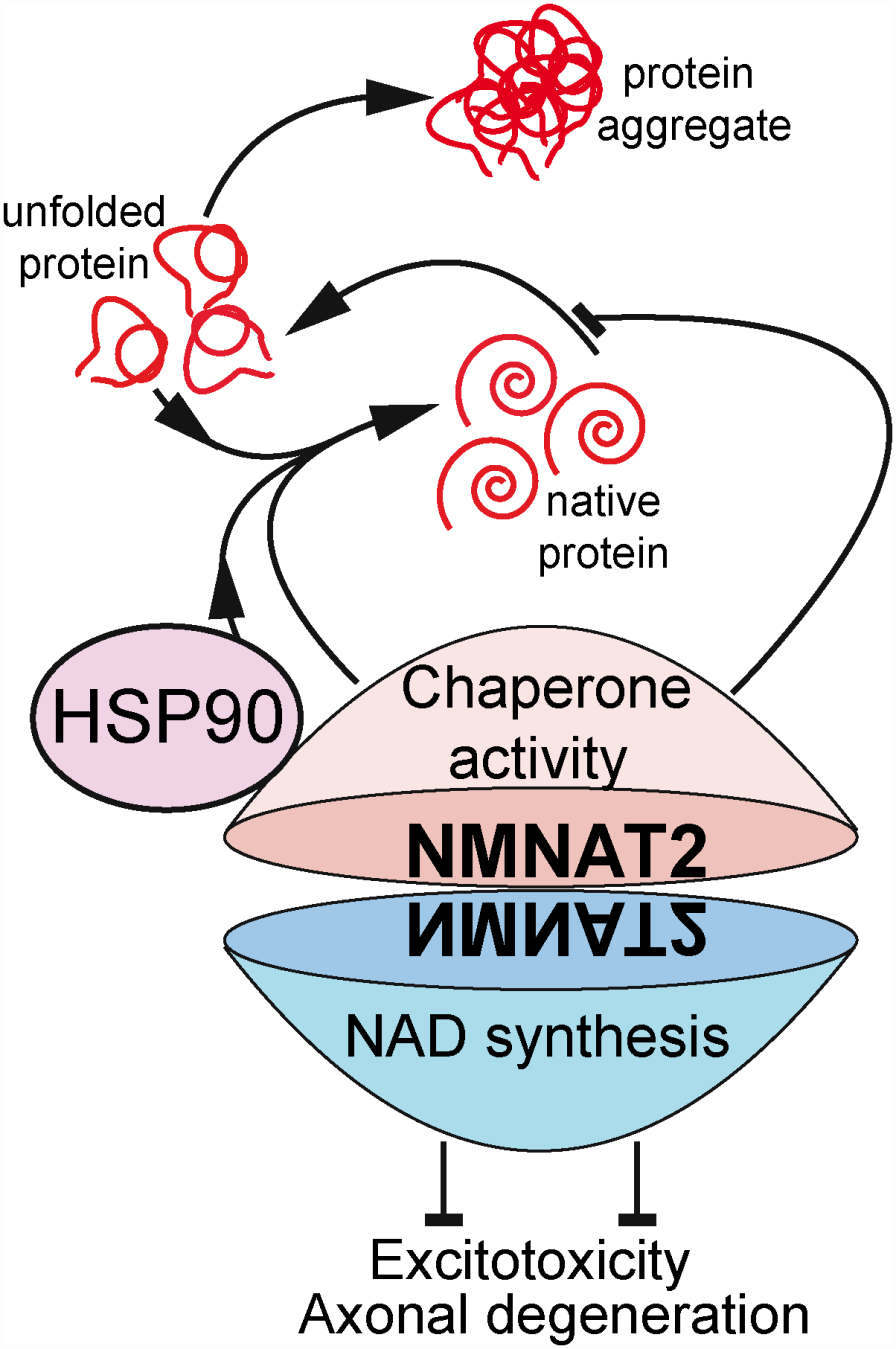
NMNAT2 dual role in axonal and neuronal maintenance. NMNAT2 acts as a molecular chaperone that cooperates with HSP90 to refold denatured proteins (foldase activity) and independently prevents protein unfolding (holdase activity). In addition, NMNAT2’s NAD synthase activity protects against excitotoxicity and axonal injury.

The work by Ali et al. opens exciting perspectives for future investigations to deeply understand the role of NMNAT2. A key question to answer is which are the NMNAT2 client proteins in physiological conditions? Identifying those client proteins will give important hints about the mechanisms by which NMNAT2 helps to maintain neuronal health and could unveil an as-yet-unknown Achilles heel that compromises neuronal viability. Furthermore, the specific contribution of the chaperone activity in vivo is currently unclear. Genome editing approaches in mice would be ideal to test the full relevance of NMNAT2’s chaperone activity and to investigate if it is necessarily essential in the brain. It would be fascinating to study neuronal, axonal, and synaptic dysfunction in conditional mouse models lacking NMNAT2 in specific neuronal populations without compromising mouse viability. Certainly, the role of NMNAT2 could be very important in neurodegeneration disorders. The sequestration of α-synuclein in Lewy bodies has been hypothesized to have pathological effects as a consequence of α-synuclein depletion [2]; perhaps the trapping of NMNAT2 found in insoluble fractions in the brain of AD patients restrains NMNAT2 from acting as a chaperone. We will have to stay tuned for future experiments to fully understand the function of NMNAT2 in health and disease.

## Abbreviations

AD: Alzheimer disease
CSP- α: Cysteine String Protein-α
dNMNAT: *Drosophila* NMNAT
HSP90: heat shock protein 90
KO: knock-out
NAD+: nicotinamide adenine dinucleotide
NMNAT1: nicotinamide mononucleotide adenylyltransferase 1
p-hTau: proteotoxic hyperphosphorylated human Tau
SCA1: spinocerebellar ataxia 1
WldS: Wallerian degeneration slow
